# Metabolic syndrome and its components reduce coronary collateralization in chronic total occlusion: An observational study

**DOI:** 10.1186/s12933-021-01297-4

**Published:** 2021-05-10

**Authors:** Tong Liu, Zheng Wu, Jinghua Liu, Yun Lv, Wenzheng Li

**Affiliations:** Department of Cardiology, Beijing Anzhen Hospital, Beijing Institute of Heart, Lung and Blood Vessel Diseases, Capital Medical University, No. 2 Anzhen Street, Chaoyang District, Beijing, 100029 China

**Keywords:** Chronic total occlusion, Metabolic syndrome, Coronary collateralization, Components, Scoring system

## Abstract

**Background:**

Metabolic syndrome (MetS) is an independent risk factor for the incidence of cardiovascular diseases. We investigated whether or to what extent MetS and its components was associated with coronary collateralization (CC) in chronic total occlusion (CTO).

**Methods:**

This study involved 1653 inpatients with CTO. Data on demographic and clinical characteristics were collected by cardiovascular doctors. The CC condition was defined by the Rentrop scoring system. Subgroup analysis, mixed model regression analysis, scoring systems and receiver operating characteristic (ROC) curve analysis were performed.

**Results:**

Overall, 1653 inpatients were assigned to the poor CC group (n = 355) and good CC group (n = 1298) with or without MetS. Compared to the good CCs, the incidence of MetS was higher among the poor CCs for all patients. Poor collateralization was present in 7.6%, 14.2%, 19.3%, 18.2%, 35.6% and 51.1% of the six groups who met the diagnostic criteria of MetS 0, 1, 2, 3, 4 and 5 times, respectively. For multivariable logistic regression, quartiles of BMI remained the risk factors for CC growth in all subgroups (adjusted OR = 1.755, 95% CI 1.510–2.038, *P* < 0.001 all patients; adjusted OR = 1.897, 95% CI 1.458–2.467, *P* < 0.001 non-MetS; and adjusted OR = 1.814, 95% CI 1.482–2.220, *P* < 0.001 MetS). After adjustment for potential confounding factors, MetS was an independent risk factor for CC growth in several models. Assigning a score of one for each component, the AUCs were 0.629 (95% CI 0.595–0.662) in all patients, 0.656 (95% CI 0.614–0.699) in MetS patients and 0.569 (95% CI 0.517–0.621) in non-MetS patients by receiver operating characteristic analysis.

**Conclusions:**

MetS, especially body mass index, confers a greater risk of CC formation in CTO. The value of scoring systems should be explored further for CTO.

## Background

Chronic total occlusion (CTO) is defined as grade 0 thrombolysis in myocardial infarction (TIMI) flow for more than 3 months and is frequently encountered during coronary angiography in patients with coronary artery disease (CAD) [1]. Coronary collaterals (CCs) serve as conduits that bridge occluded coronary arteries supplied by epicardial or septal arteries, which provide an alternative source of blood supply to a myocardium subtended by an occluded vessel [2]. Good CCs can relieve angina, reduce infarct size, protect heart function and decrease mortality. In addition, guidewires easily cross and achieve recanalization effectively for good CCs [3].

Epidemiological data frequently demonstrate that metabolic syndrome (MetS) is increasingly prevalent and represents an important risk factor for cardiovascular diseases, which are characterized by a cluster of risk components, including abdominal obesity, hyperglycaemia, dyslipidaemia and hypertension [4]. Several groups have reported that a higher number of metabolic syndrome components is correlated with serious CAD [5, 6]. Vasculogenesis, angiogenesis and arteriogenesis are three important processes for the formation of CCs, which are influenced by signalling, transcriptional control, soluble mediators and their receptors, biomechanical forces and hypoxia [7]. Abdominal obesity and dyslipidaemia indicate vascular endothelial dysfunction, which impairs the processes of CCs [8]. Shen Y et al. revealed that type 2 diabetes mellitus (T2DM) adversely affects coronary collateral development through multiple cellular mechanisms [9]. There is an inverse relationship between high blood pressure (BP) and the maturation of CCs [10]. Nevertheless, this result remains controversial, and the exact mechanism is still unknown.

Despite the evidence indicating the association between MetS and CCs [11], the value of MetS as a predictive biomarker for CCs remains controversial in CTO patients [12], and the prognostic power of MetS components is also unknown. Therefore, we investigated the association of MetS and its components with CC development and deduced the usefulness of MetS for the diagnosis and risk assessment of CCs in clinical practice.

## Methods

### Study population

**Fig. 1:**
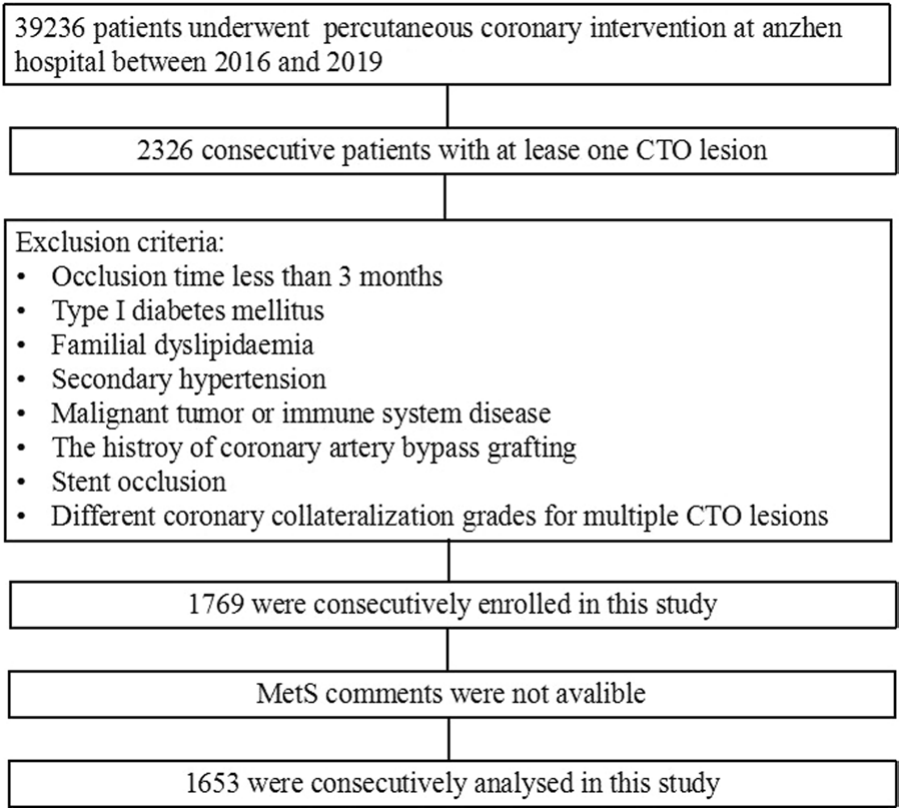
Population flowchart of enrolled patients

This was an observational cohort study conducted at the Beijing Anzhen Hospital of Capital Medical University between January 2016 and December 2019. Consecutive patients with CTO who underwent coronary angiography were included. Overall, 1653 inpatients were assigned to the MetS (n = 897) and non-MetS (control, n = 812) groups. The major exclusion criteria included the following: occlusion time less than 3 months, type I diabetes mellitus, familial dyslipidaemia, secondary hypertension, malignant tumour, immune system disease, history of coronary artery bypass grafting, stent occlusion and different coronary collateralization grades for multiple CTO lesions. The detailed recruitment process is depicted in Fig. 1.

CTO was defined as follows: 1) an occlusion lasting for more than 3 months based on the first onset of angina pectoris, previous angiogram findings and previous infarction; and 2) TIMI grade 0. According to the criteria of the American National Cholesterol Education Program, MetS was defined as the presence of three or more of the following criteria: body mass index (BMI) > 30 kg/m^2^, high-density lipoprotein (HDL) < 50 mg/dL among women and < 40 mg/dL among men, fasting plasma triglycerides (TG) ≥ 150 mg/dL, systolic blood pressure (SBP) ≥ 130 mmHg, diastolic blood pressure (DBP) ≥ 85 mmHg, fasting plasma glucose (FPG) ≥ 100 mg/dL or previously diagnosed type 2 diabetes (T2DM) [13]. All participants underwent bilateral coronary angiography, which was conducted by an experienced team of cardiologists. The degree of coronary collateralization was visually estimated using the Rentrop scoring system. Good collateralization was defined as graded Rentrop 2 or 3, and poor collateralization was defined as graded Rentrop 0 or 1 [14]. The study protocol was approved by the Institutional Review Board of The Anzhen Hospital, Beijing, China.

### Procedure

Data regarding the sex, age, medical history, smoking status, BMI, BP, heart rate (HR), complete blood count, serum cholesterol level and homocysteine concentration were collected for all participants. The diagnostic criteria for classic risk factors, including hypertension (HT) [15] and T2DM [16], were based on authoritative international guidelines. Blood samples were drawn from all participants and analysed by an automated biochemical analyser, which included creatinine, lipid profiles, glucose and glycated albumin (GA).

### Statistical analyses

Continuous variables are reported as the means ± standard deviations for normally distributed data or medians and quartiles (quartile 1; quartile 3) for non-normally distributed data, and they were compared using Student’s t test for normally distributed data or the Mann–Whitney U test for non-normally distributed data. The Kolmogorov–Smirnov test was used to check normality. Discrete variables are expressed as frequencies and percentages and were compared using the chi-square test. Univariable and multivariable logistic regression analyses were performed to detect the relationship between coronary collateralization and MetS. In multivariate analysis, odds ratios (ORs) and 95% confidence intervals (CIs) for coronary collateralization were calculated using the logistic regression model after adjusting for potential confounding variables. There were eight mixed regression models, which were adjusted for age, sex, heart rate, renal disease, former smoker, current smoker, prior myocardial infarction, stroke, history of percutaneous coronary intervention, white blood cell count, homocysteine level, platelet count, creatinine level, eGFR level, glycated albumin level, uric acid level, ventricular ejection fraction, left ventricular end-diastolic dimension, elevated left ventricular end-systolic dimension, body mass index, elevated blood pressure, elevated fasting glucose, reduced high-density lipoprotein cholesterol, elevated triglycerides, diuretics, ACE inhibitors/ARBs, β-blockers, CCBs and hypoglycaemic agents. Generalized estimating equations were used to explore the impact of multiple CTO lesions. To verify the robustness of our results, subgroup analyses were performed to explore the association between the number of total MetS components and poor collateralization. These predictors of metabolic syndrome components were assigned corresponding points based on their regression coefficient, thereby generating a scoring system. Receiver operating characteristic (ROC) curves were constructed, and the areas under the curves (AUCs) were calculated to assess the discriminatory power of MetS for the scoring system. A two-sided *p*-value < 0.05 was considered statistically significant. The statistical computations were performed using SPSS software, version 23.0 (IBM Corp., Armonk, NY, USA).

## Results

### Baseline characteristics

For poor CCs, there were 222 patients (25.8%) among the MetS and 133 patients (16.8%) among the non-MetS groups. Compared to good CCs, the incidence of HT and T2DM was significantly higher among the poor CCs for all patients (all *P* < 0.001). There were no significant differences in age or sex between the MetS subgroup and the non-MetS subgroup. Compared to good CCs, BMI was higher in poor CCs for both MetS and non-MetS patients (*P* < 0.001). The DBP, TG and GA levels were more elevated in patients with poor CCs for all patients (all *P* < 0.001). Similarly, poor CCs showed significantly higher SBP, FBG and TC levels than good CCs in all patients and in the MetS subgroup (all *P* < 0.05). The RCA CTO lesion was predominant in all patients. Furthermore, except for ACE inhibitors/ARBs and hypoglycaemic agents, there were no significant differences in medical treatments in all patients and in the subgroups (Table 1).

**Table 1:** clinical characteristics in MetS and non-MetS patients with poor and good collateralization

Variables	Overall			MetS			non-MetS		
	Poor collateralization n = 355	Good collateralization n = 1298	*P* value	Poor collateralization n = 222	Good collateralization n = 640	*P* value	Poor collateralization n = 133	Good collateralization n = 658	*P* value
Male, n(%)	294 (82.8)	1074 (82.7)	0.974	174 (78.4)	508 (79.4)	0.753	120 (90.2)	566 (86.0)	0.192
Age, years	57.7 ± 10.5	59.1 ± 10.3	0.022	57.2 ± 10.6	58.7 ± 10.2	0.057	58.6 ± 10.4	59.6 ± 10.3	0.346
BMI, Kg/m^2^	28.1 ± 3.7	26.0 ± 3.1	< 0.001	28.9 ± 4.0	26.9 ± 3.1	< 0.001	26.9 ± 2.8	25.2 ± 2.8	< 0.001
HT, n(%)	255 (71.8)	816 (62.9)	0.002	182 (82.0)	497 (77.7)	0.174	73 (54.9)	319 (48.5)	0.178
T2DM, n(%)	156 (43.9)	414 (31.9)	< 0.001	118 (53.2)	285 (44.5)	0.027	38 (28.6)	129 (19.6)	0.021
Renal disease, n(%)	10 (2.8)	25 (1.9)	0.302	8 (3.6)	13 (2.0)	0.190	2 (1.5)	12 (1.8)	0.799
Metabolic syndrome, n(%)	222 (62.5)	640 (49.3)	< 0.001	–	–	–	–	–	–
Former smoker, n(%)	56 (15.8)	211 (16.3)	0.827	32 (14.4)	98 (15.3)	0.747	24 (18.0)	113 (17.2)	0.809
Current smoker, n(%)	146 (41.1)	540 (41.6)	0.872	80 (36.0)	267 (41.7)	0.137	66 (49.6)	273 (41.5)	0.197
Prior MI, n(%)	102 (28.7)	359 (27.7)	0.689	63 (28.4)	189 (29.5)	0.745	39 (29.3)	170 (25.8)	0.405
Stroke, n(%)	20 (5.6)	89 (6.9)	0.411	14 (6.3)	49 (7.7)	0.505	6 (4.5)	40 (6.1)	0.481
History of PCI, n(%)	103 (29.0)	386 (29.7)	0.791	69 (31.1)	211 (33.0)	0.605	34 (25.6)	175 (26.6)	0.806
SBP, mmHg	132.6 ± 16.1	127.3 ± 16.1	< 0.001	135.1 ± 15.5	128.6 ± 16.3	< 0.001	128.3 ± 16.4	126.0 ± 15.7	0.117
DBP, mmHg	79.5 ± 11.8	75.7 ± 10.6	< 0.001	81.0 ± 12.0	76.3 ± 10.6	< 0.001	77.2 ± 11.0	75.0 ± 10.5	0.033
HR, bpm	71.7 ± 9.4	72.0 ± 10.5	0.558	71.8 ± 9.4	72.7 ± 10.5	0.309	71.4 ± 9.3	71.4 ± 10.5	0.967
FBG, mmol/L	6.2 (5.1, 8.1)	5.7 (5.1, 7.2)	< 0.001	6.9 (5.6, 8.1)	6.4 (5.5, 8.1)	0.033	5.4 (4.9, 6.4)	5.3 (4.9, 5.9)	0.163
TG, mmol/L	1.6 (1.2,2.4)	1.4 (1.0,2.0)	< 0.001	2.0 (1.6, 2.8)	1.9 (1.3, 2.5)	0.002	1.2 (1.1, 1.6)	1.1 (0.9, 1.4)	< 0.001
TC, mmol/L	4.1 ± 1.3	3.8 ± 1.0	0.001	4.1 ± 1.5	3.8 ± 1.1	0.004	4.0 ± 1.0	3.8 ± 1.0	0.109
LDL-C, mmol/L	2.3 ± 1.0	2.2 ± 0.9	0.189	2.3 ± 1.1	2.2 ± 0.9	0.319	2.4 ± 0.9	2.3 ± 0.9	0.257
HDL-C, mmol/L	1.0 (0.8, 1.2)	1.0 (0.9, 1.2)	0.007	0.9 (0.8, 1.0)	0.9(0.8, 1.0)	0.545	1.1(0.9, 1.2)	1.1(1.0, 1.3)	0.489
WBC, 10^12^/L	7.1 ± 1.8	7.0 ± 1.8	0.179	7.0 ± 1.8	7.1 ± 1.8	0.526	7.2 ± 1.8	6.8 ± 1.8	0.052
HGB, g/L	144 (133,153)	142 (132,152)	0.149	143 (133, 154)	142 (132, 152)	0.293	144 (134,152)	142 (132, 152)	0.345
PLT, 10^9^/L	217.2 ± 54.5	218.4 ± 57.0	0.719	219.0 ± 54.8	219.4 ± 57.0	0.803	214.1 ± 54.1	217.4 ± 57.1	0.546
Cr, μmol/L	78.5 ± 21.5	77.0 ± 33.1	0.418	79.3 ± 23.7	77.9 ± 31.4	0.562	77.3 ± 17.2	76.1 ± 34.6	0.712
UA, μmol/L	361.2 (310.9, 418.2)	350.7 (299.2, 419.0)	0.186	364.1 (311.0, 425.3)	363.6 (307.5, 432.7)	0.778	357.5 (309.2, 410.9)	341.1 (291.3, 406.1)	0.072
eGFR, mL/(min·1.73 m2)	92.8 ± 17.5	93.3 ± 17.0	0.657	92.0 ± 18.9	92.6 ± 17.8	0.676	94.3 ± 14.8	94.0 ± 16.1	0.834
HCY, μmol/L	16.2 ± 8.9	16.2 ± 10.0	0.930	15.7 ± 8.1	15.8 ± 9.6	0.878	17.0 ± 10.1	16.6 ± 10.3	0.715
GA, %	15.3 (13.8, 17.8)	14.5 (13.3, 16.6)	< 0.001	15.6 (14.0, 18.0)	14.9 (13.4,18.3)	0.023	14.7 (13.5, 16.7)	14.2 (13.3, 15.8)	0.039
LVEF, %	56.9 ± 7.8	56.1 ± 7.8	0.134	57.0 ± 7.2	56.6 ± 7.7	0.513	56.8 ± 8.6	55.8 ± 7.9	0.212
LVEDd, mm	50.6 ± 5.2	51.0 ± 5.2	0.249	50.1 ± 5.0	50.8 ± 5.2	0.084	51.5 ± 5.5	51.2 ± 5.2	0.502
LVEDs, mm	35.3 ± 6.0	35.8 ± 5.8	0.164	35.0 ± 5.4	35.5 ± 5.7	0.254	35.8 ± 6.8	36.1 ± 5.8	0.644
Medication, n (%)									
Antiplatelet	355 (100)	1298 (100)	1	222 (100)	640 (100)	1	133 (100)	658 (100)	1
Statins	355 (100)	1298 (100)	1	222 (100)	640 (100)	1	133 (100)	658 (100)	1
Diuretics	45 (12.4)	158 (12.2)	0.798	33 (14.9)	84 (13.1)	0.514	12 (9.0)	74 (11.2)	0.452
ACE inhibitors/ARBs	150 (42.3)	471 (36.3)	0.040	107 (48.2)	276 (43.1)	0.190	43 (32.3)	195 (29.6)	0.536
β-blockers	236 (66.5)	846 (65.2)	0.648	148 (66.7)	446 (69.7)	0.402	88 (66.2)	400 (60.8)	0.245
CCBs	111 (31.3)	369 (28.4)	0.296	71 (31.3)	221 (34.6)	0.489	40 (30.1)	148 (22.5)	0.061
Hypoglycaemic agents	107 (30.1)	320 (24.7)	0.036	83 (37.4)	216 (33.8)	0.327	24 (18.0)	104 (15.3)	0.522
Target vessel, n (%)									
RCA	167 (47.0)	576 (44.4)	0.371	104 (46.8)	288 (45.0)	0.634	63 (47.4)	288 (43.8)	0.446
LAD	132 (37.2)	491 (37.8)	0.824	89 (40.1)	227 (35.5)	0.218	43 (32.3)	264 (40.1)	0.093
LCX	63 (17.7)	240 (18.5)	0.748	33 (14.9)	130 (20.3)	0.074	30 (22.6)	110 (16.7)	0.109
Elevated BMI, n (%)	88 (24.8)	136 (10.5)	< 0.001	83 (37.4)	117 (18.3)	< 0.001	5 (3.8)	19 (2.9)	0.593
Elevated BP,n (%)	293 (82.5)	957 (73.7)	0.001	208 (97.3)	559 (87.3)	0.009	85 (63.9)	398 (60.5)	0.460
Elevated FBG, n (%)	238 (67.0)	751 (57.9)	0.002	181 (81.5)	516 (80.6)	0.767	57 (42.9)	235 (35.7)	0.119
Reduced HDL-C, n (%)	247 (69.6)	837 (64.5)	0.073	195 (87.8)	580 (90.6)	0.235	52 (39.1)	257 (39.1)	0.993
Elevated TG, n (%)	166 (46.8)	447 (34.4)	< 0.001	147 (66.2)	376 (58.8)	0.050	19 (14.3)	71 (10.8)	0.247

*BMI* body mass index, *HT *Hypertension, *T2DM* type 2 diabetes mellitus, *Prior MI *Prior myocardial infarction, *History of PCI* history of percutaneous coronary intervention, *History of CABG *history of coronary artery bypass grafting, *SBP* systolic blood pressure, D*BP* diastolic blood pressure, *HR* heart rate, *FBG* Fasting blood glucose, *TG* Triglycerides, *TC *Total cholesterol, *LDL-C* low-density lipoprotein cholesterol, *HDL-C* high-density lipoprotein cholesterol, *WBC* white blood cell, *HGB *hemoglobin, *PLT* Platelet, *Cr *creatinine, *UA *Uric acid, *HCY *Homocysteine, *GA *Glycated albumin, *LVEF *ventricular ejection fraction, *LVEDd* left ventricular end-diastolic dimension, *LVEDs*left ventricular end-systolic dimension, *ACE inhibitors/ARBs* Angiotensin-Converting Enzyme Inhibitors/Angiotensin Receptor Blockers, *CCBs *calcium channel blockers, *RCA* Right coronary artery, *LAD* Left anterior descending, *LCX *left circumflex coronary artery

### MetS number and clinical characteristics

**Fig. 2:**
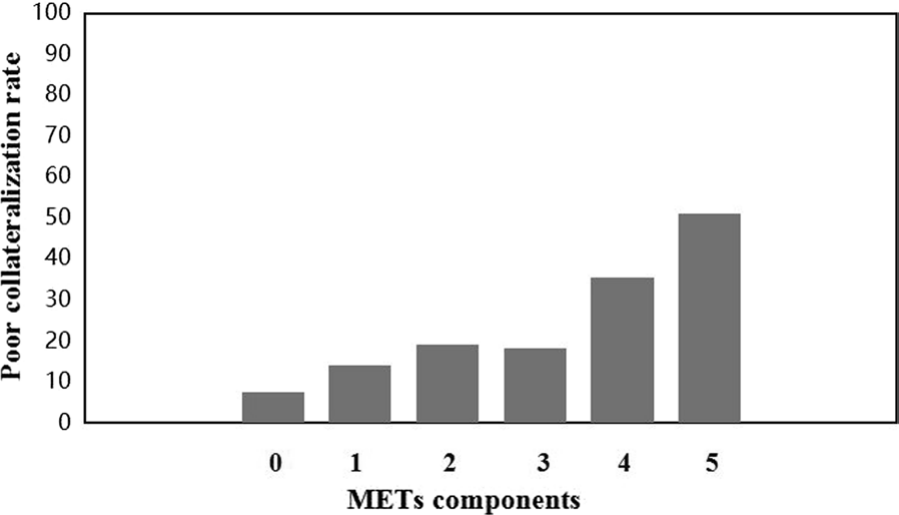
Poor collateralization of metabolic syndrome components

Individuals were divided into six groups as follows: 52 (3.1%), 280 (16.9%), 459 (27.8%), 531 (32.1%), 286 (17.3%) and 45 (2.7%) patients who met the diagnostic criteria for 0, 1, 2, 3, 4 and 5 MetS components, respectively (Fig. 2). Poor collateralization was present in 7.6%, 14.2%, 19.3%, 18.2%, 35.6% and 51.1% of the patients who met the diagnostic criteria for 0, 1, 2, 3, 4 and 5 MetS components, and these differences were significant (*P* < 0.05, Table 2). The prevalence of males was the highest in the 0 group. Except for LDL-C and LVEF, there were significant differences in the variables among the six groups (all *P* < 0.001). With regard to MetS components, BMI, HT, T2DM, BP and TG increased with the increase in traits. For comparison among groups, the difference was the greatest in BMI (*P* < 0.001, Table 2).

**Table 2:** Baseline characteristics of the number of MetS

Variables	The number of the presence of MetS							
	0 n = 52	1 n = 280	2 n = 459	3 n = 531	4 n = 286	5 n = 45	*P* value	*P* < 0.05
Male, n(%)	51 (98.0)	251 (89.6)	384 (83.6)	421 (79.2)	224 (78.3)	38 (82.2)	<0.001	a,b,c,d,e,f,g,h
Age, years	59.3 ± 9.0	59.0 ± 10.6	59.7 ± 10.3	59.1 ± 9.9	57.6 ± 11.1	54.3 ± 10.3	0.005	e,i,k,l,m,n
BMI, Kg/m2	24.8 ± 2.7	25.1 ± 2.8	25.8 ± 2.8	26.5 ± 2.9	28.2 ± 3.7	32.4 ± 2.0	< 0.001	b,c,d,e,f,g,h.i,j,k,l,m,n,o
HT, n(%)	0 (0)	120 (42.8)	272 (59.2)	388 (73.0)	250 (87.4)	41 (91.1)	< 0.001	a,b,c,d,e,f,g,h,j,k,l,m,n
T2DM, n(%)	0 (0)	31 (11.0)	136 (29.6)	225 (42.3)	155 (54.1)	23 (51.1)	< 0.001	a,b,c,d,e,f,g,h,j,k,l,m
Poor CC, n(%)	4 (7.6)	40 (14.2)	89 (19.3)	97 (18.2)	102 (35.6)	23 (51.1)	< 0.001	d,e,h,k,m,n,o
SBP, mmHg	118.2 ± 8.7	126.6 ± 16.3	127.2 ± 16.0	129.3 ± 16.2	132.3 ± 16.9	129.5 ± 12.8	< 0.001	a,b,c,d,e,g,h,j,k,m
DBP, mmHg	71.0 ± 7.0	75.7 ± 11.3	75.7 ± 10.4	76.9 ± 11.0	78.4 ± 11.6	79.3 ± 10.4	< 0.001	a,b,c,d,e,h,i,k,l
HR, bpm	67.3 ± 7.0	71.1 ± 10.5	72.1 ± 10.4	72.3 ± 9.7	73.0 ± 11.4	70.7 ± 8.1	0.005	a,b,c,d,e,h
FBG, mmol/L	5.1 (4.6, 5.4)	5.2 (4.8, 5.4)	5.4 (5.0, 6.6)	6.2 (5.3, 8.0)	6.9 (5.9, 8.5)	7.6 (6.5, 9.6)	< 0.001	b,c,d,e,f,g,h,i,j,k,l,m,n,o
TG, mmol/L	1.2 (0.9, 1.3)	1.1 (0.8, 1.3)	1.2 (1.0, 1.6)	1.6 (1.2, 2.3)	2.2 (1.8, 2.9)	2.5 (2.1, 3.9)	< 0.001	b,c,d,e,f,g,h,i,j,k,l,m,n,o
GA, %	13.8 (13.1, 15.4)	14.1 (13.2, 15.9)	14.3 (13.4, 16.2)	14.7 (13.4, 17.4)	15.8 (13.8, 19.1)	15.9 (14.2, 19.0)	<0.001	b,c,d,e,f,g,h,i,k,l,m
TC, mmol/L	3.8 ± 0.8	3.9 ± 1.0	3.8 ± 1.1	3.8 ± 1.1	4.0 ± 1.3	4.2 ± 1.0	0.036	k,l,m,n
LDL-C, mmol/L	2.2 ± 0.8	2.3 ± 0.9	2.3 ± 1.0	2.2 ± 0.9	2.3 ± 1.0	2.3 ± 0.8	0.363	
HDL-C, mmol/L	1.2 (1.1, 1.3)	1.2 (1.1, 1.3)	1.0 (1.0, 1.2)	0.9 (0.8, 1.0)	0.9 (0.8, 1.0)	0.9 (0.8, 1.0)	< 0.001	b,c,d,e,f,g,h,i,j,k,l,m,n
LVEF, %	54.4 ± 10.3	56.1 ± 8.1	56.0 ± 7.6	56.7 ± 7.4	56.6 ± 7.9	56.6 ± 7.0	0.419	

a:0vs1,b:0vs2,c:0vs3,d:0vs4,e:0vs5,f:1vs2,g:1vs3,h:1vs4,i:1vs5,g:2vs3,k:2vs4,l:2vs5,m:3vs4,n:3vs5,o:4vs5

*METs *Metabolic Syndrome, *BMI* body mass index, *HT *Hypertension, *T2DM* type 2 diabetes mellitus,* CC *Coronary Collateralization, *SBP* systolic blood pressure, *DBP* diastolic blood pressure, *HR* heart rate, *FBG *Fasting blood glucose, *TG* Triglycerides, *TC *Total cholesterol, *LDL-C* low-density lipoprotein cholesterol; *HDL-C* high-density lipoprotein cholesterol, *GA *Glycated albumin, *LVEF *ventricular ejection fraction

### MetS components and coronary collateralization

After adjustment for several potential risk factors, such as sex, age, smoking status, and prior myocardial infarction, the quartiles of BMI (adjusted OR = 1.728, 95% CI 1.518–1.967, *P* < 0.001), TG (adjusted OR = 1.278, 95% CI 1.125–1.451, *P* < 0.001), SBP (adjusted OR = 1.267, 95% CI 1.088–1.474, *P* = 0.002) and DBP (adjusted OR = 1.202, 95% CI 1.010–1.430, *P* = 0.038) remained independent factors of poor CCs. Patients with T2DM had a significantly increased risk of poor CCs compared to those with no T2DM in all groups (adjusted OR = 1.664, 95% CI 1.053–2.629, *P* < 0.001). Compared to the first BMI quartile, the ORs of incident poor CCs were 4.852 (95% CI 2.934–8.024), 3.594 (95% CI 2.324–5.560) and 1.464 (95% CI 1.010–2.121) for the second, third and fourth BMI quartiles, respectively, after adjusting for potential risk factors (Table 3).

**Table 3:** Impact of MetS components on coronary collateral growth in patients with and without MetS

Variables	Quartiles of components		Non-MetS			MetS			All		
	Range	n	Good/Poor	OR (95%CI)	*P* Value	Good/Poor	OR (95%CI)	*P* Value	Good/Poor	OR (95%CI)	*P* Value
HT, n(%)	–	1071	73/319	1.386 (0.758–2.534)	0.289	182/497	1.478 (0.881–2.481)	0.139	255/816	1.179 (0.818–1.699)	0.377
T2DM, n(%)	–	570	38/129	2.292 (0.886–5.929)	0.087	118/285	1.718 (0.988–2.987)	0.055	156/414	1.664 (1.053–2.629)	0.029
BMI, Kg/m2	Per quartile	1653	140/672	1.897 (1.458–2.467)	< 0.001	230/667	1.814 (1.482–2.220)	< 0.001	370/1339	1.755 (1.510–2.038)	< 0.001
	Q1 ≤ 24.33	412	19/225	–	–	21/137	–	–	40/362	–	–
	24.33 < Q2 ≤ 26.29	415	30/187	6.413 (2.679–15.354)	< 0.001	30/168	5.424 (2.688–10.943)	< 0.001	60/355	4.852 (2.934–8.024)	< 0.001
	26.29 < Q3 ≤ 28.37	413	54/164	4.330 (1.952–9.604)	< 0.001	54/141	4.399 (2.467–7.844)	< 0.001	108/305	3.594 (2.324–5.560)	< 0.001
	28.37 < Q4	413	30/72	1.720 (0.847–3.490)	0.133	117/194	1.768 (1.085–2.940)	0.023	147/266	1.464 (1.010–2.121)	0.044
TG, mmol/L	Per quartile	1653		1.664 (1.264–2.190)	< 0.001		1.407 (1.136–1.744)	0.002	370/1339	1.371 (1.184–1.588)	< 0.001
	Q1 ≤ 1.08	412	30/275	–	–	20/87	–	–	50/362	–	–
	1.09 < Q2 ≤ 1.44	421	57/225	5.940 (2.203–16.012)	< 0.001	28/111	2.324 (1.097–4.927)	0.028	85/336	2.764 (1.694–4.510)	< 0.001
	1.44 < Q3 ≤ 2.07	410	30/119	2.093 (0.822–5.311)	0.121	71/190	2.750 (1.422–5.320)	0.003	101/309	1.609 (1.040–2.491)	0.033
	2.07 < Q4	410	16/39	2.087 (0.806–5.400)	0.129	103/252	1.149 (0.727–1.816)	0.551	119/291	1.161 (0.790–1.706)	0.447
HDL-C, mmol/L	Per quartile	1653		0.970 (0.751–1.252)	0.812		0.918 (0.741–1.137)	0.431	370/1339	1.006 (0.866–1.168)	0.937
	Q1 ≤ 0.84	414	20/85	–	–	83/226	–	–	103/311	–	–
	0.84<Q2≤0.98	408	21/99	0.893 (0.396–2.015)	0.786	69/219	0.751 (0.345–1.631)	0.469	90/318	1.092 (0.679–1.756)	0.715
	0.98<Q3 ≤ 1.16	412	38/194	1.179 (0.524–2.651)	0.691	45/135	0.882 (0.407–1.912)	0.751	83/329	1.241 (0.775–1.989)	0.368
	1.16<Q4	419	54/280	1.558 (0.794–3.055)	0.197	25/60	0.938 (0.416–2.114)	0.877	79/340	1.223 (0.761–1.965)	0.406
SBP, mmHg	Per quartile	1653		1.162 (0.858–1573)	0.333		1.362 (1.078–1.720)	0.010	370/1339	1.252 (1.049–1.496)	0.013
	Q1 ≤ 119	425	32/199	–	–	31/163	-	–	63/362	–	–
	119<Q2≤128	412	39/181	1.715 (0.631–4.664)	0.290	38/154	2.438 (1.140–5.215)	0.022	77/335	1.975 (1.121–3.478)	0.019
	128<Q3 ≤ 137	400	29/148	1.215 (0.519–2.844)	0.653	62/161	1.915 (1.016–3.610)	0.044	91/309	1.601 (0994–2.579)	0.053
	137<Q4	416	33/130	1.270 (0.546–2.956)	0.579	91/162	1.215 (0.712–2.072)	0.475	124/292	1.319 (0.862–2.019)	0.202
DBP, mmHg	Per quartile	1653		1.089 (0.806–1.471)	0.579		1.287 (1.024–1.617)	0.030	370/1339	1.202 (1.010–1.430)	0.038
	Q1 ≤ 69	381	27/168	–	–	37/149	–	–	64/317	–	–
	69<Q2≤75	424	37/191	1.439 (0.531–3.901)	0.640	37/159	1.905 (0.939–3.865)	0.074	74/350	1.769 (1.029–3.044)	0.039
	75<Q3 ≤ 82	417	33/178	1.630 (0.692–3.838)	0.288	45/161	2.711 (1.437–5.135)	0.002	78/339	1.967 (1.230–3.146)	0.005
	82^<^Q4	431	36/121	1.848 (0.796–4.292)	0.153	103/171	2.794 (1.573–4.963)	< 0.001	139/292	2.215 (1.415–3.465)	< 0.001
FBG, mmol/L	Per quartile	1709		1.018 (0.735–1.409)	0.917		1.006 (0.817–1.238)	0.955	370/1339	0.968 (0.825–1.136)	0.691
	Q1 ≤ 5.12	420	47/233	–	–	36/104	–	–	83/337	–	
	5.12<Q2≤5.76	410	38/244	1.505 (0.499–4.542)	0.468	27/101	1.047 (0.540–2.029)	0.892	65/345	0.922 (0.565–1.505)	0.745
	5.76<Q3 ≤ 7.33	411	22/101	2.354 (0.745–7.442)	0.145	68/220	1.193 (0.614–2.320)	0.603	90/321	1.305 (0.787–2.163)	0.302
	7.33<Q4	412	26/80	1.654 (0.577–4.745)	0.349	91/215	1.042 (0.628–1.730)	0.873	117/295	1.156 (0.748–1.786)	0.514

*OR *odds ratio, *CI*confdence interval, *MetS *Metabolic Syndrome, *BMI* body mass index, *HT* Hypertension, *T2DM* type 2 diabetes mellitus, *HDL-C *high-density lipoprotein cholesterol, *TG *Triglycerides, *FBG *Fasting blood glucose, *SBP* systolic blood pressure, *DBP* diastolic blood pressure

^a^Multiple adjustment for adjusted for age, sex, heart rate, renal disease, former smoker, current smoker, prior myocardial infarction, stroke, history of percutaneous coronary intervention, white blood cell, homocysteine, platelet, creatinine, eGFR, glycated albumin, uric acid, ventricular ejection fraction; left ventricular end-diastolic dimension; left ventricular end-systolic dimension, Diuretics, ACE inhibitors/ARBs,β-blockers, CCBs, hypoglycemic agents

### MetS and coronary collateralization

Table 4 shows the results of multivariate logistic regression for the association between the incidence of poor CCs and MetS. There were eight models after adjusting for age, sex, heart rate, renal disease, former smoker, current smoker, prior myocardial infarction, stroke, history of percutaneous coronary intervention, white blood cell count, homocysteine level, platelet count, creatinine level, eGFR level, glycated albumin level, uric acid level, ventricular ejection fraction, left ventricular end-diastolic dimension, elevated left ventricular end-systolic dimension, elevated body mass index, elevated blood pressure, elevated fasting glucose, reduced high-density lipoprotein cholesterol, elevated triglycerides, diuretics, ACE inhibitors/ARBs, β-blockers, CCBs and hypoglycaemic agents. The ORs were 1.690, 1.695, 1.709, 1.765, 1.721, 1.882, 1.892 and 1.921 for MetS in models 1, 2, 3, 4, 5, 6, 7 and 8, respectively (all *P* < 0.05). After completing the generalized estimating equations procedure, the ORs did not change significantly (Table 4).

**Table 4:** Odds ratio and 95% confdence interval for coronary collateral growth

Models	MetS		MetS after GEE	
	*OR *(95%CI)	*P* Value	*OR *(95%CI)	*P* Value
Model 1	1.690 (1.326–2.155)	< 0.001	1.690 (1.328–2.152)	< 0.001
Model 2	1.695 (1.328–2.162)	< 0.001	1.695 (1.331–2.158)	< 0.001
Model 3	1.709 (1.338–2.183)	< 0.001	1.709 (1.340–2.179)	< 0.001
Model 4	1.765 (1.337–2.331)	< 0.001	1.765 (1.335–2.333)	< 0.001
Model 5	1.721 (1.273–2.325)	0.001	1.721 (1.276–2.320)	< 0.001
Model 6	1.882 (1.185–2.989)	0.007	1.862 (1.093–3.172)	0.022
Model 7	1.892 (1.096–3.268)	0.022	1.892 (1.107–3.236)	0.020
Model 8	1.921 (1.109–3.326)	0.020	1.921 (1.120–3.292)	0.018

Model 1: adjusted for age, sex

Model 2: adjusted for age, sex, heart rate, renal disease, former smoker, current smoker

Model 3: adjusted for age, sex, heart rate, renal disease, former smoker, current smoker, prior myocardial infarction, stroke, history of percutaneous coronary intervention

Model 4: adjusted for age, sex, heart rate, renal disease, former smoker, current smoker, prior myocardial infarction, stroke, history of percutaneous coronary intervention, white blood cell, homocysteine, platelet, creatinine, eGFR, glycated albumin, uric acid

Model 5: adjusted for age, sex, heart rate, renal disease, former smoker, current smoker, prior myocardial infarction, stroke, history of percutaneous coronary intervention, white blood cell, homocysteine, platelet, creatinine, eGFR, glycated albumin, uric acid,ventricular ejection fraction; left ventricular end-diastolic dimension; left ventricular end-systolic dimension;

Model 6: adjusted for elevated body mass index, elevated blood pressure, elevated fasting glucose, reduced high-density lipoprotein cholesterol,elevated triglycerides;

Model 7: adjusted for age, sex, heart rate, renal disease, former smoker, current smoker, prior myocardial infarction, stroke, history of percutaneous coronary intervention, white blood cell, homocysteine, platelet, creatinine, eGFR, glycated albumin, uric acid, ventricular ejection fraction; left ventricular end-diastolic dimension; left ventricular end-systolic dimension elevated,body mass index, elevated blood pressure, elevated fasting glucose, reduced high-density lipoprotein cholesterol, elevated triglycerides

Model 8: adjusted for age, sex, heart rate, renal disease, former smoker, current smoker, prior myocardial infarction, stroke, history of percutaneous coronary intervention, white blood cell, homocysteine, platelet, creatinine, eGFR, glycated albumin, uric acid, ventricular ejection fraction; left ventricular end-diastolic dimension; left ventricular end-systolic dimension elevated, body mass index, elevated blood pressure, elevated fasting glucose, reduced high-density lipoprotein cholesterol, elevated triglycerides, Diuretics, ACE inhibitors/ARBs, β-blockers, CCBs, hypoglycaemic agents

*GEE *generalized estimating equation, *OR* odd ratio, *MetS* metabolic syndrome

### Scoring system of MetS and ROC

**Fig. 3:**
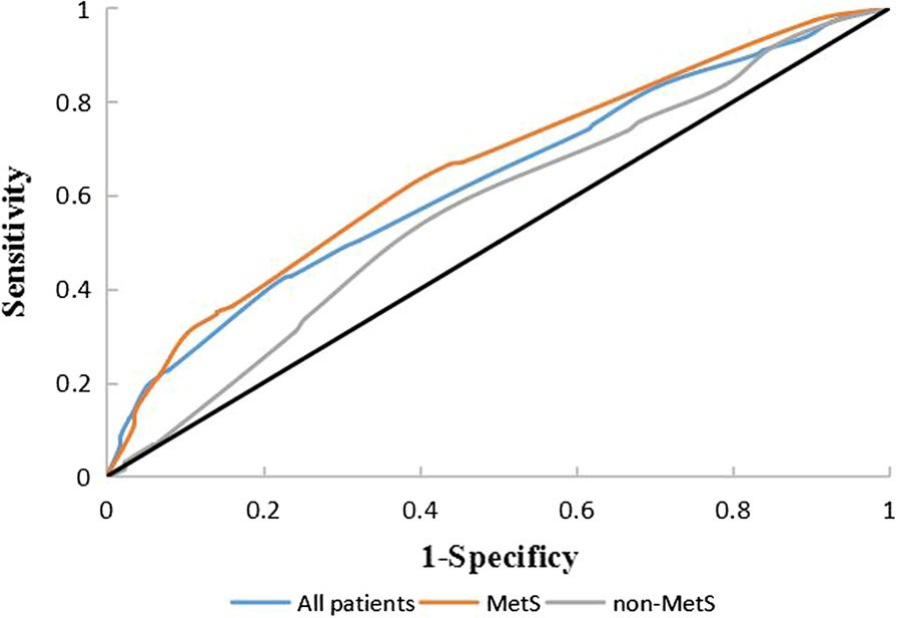
ROC curve analysis of the scoring system for the prediction of poor collateralization

Based on the regression coefficient, a point was assigned to MetS components and subsequently summed to obtain a total difficulty score after adjusting for covariates as follows: elevated BMI was 9, elevated BP was 6, elevated FBG was 3, reduced HDL was 1, and elevated TG was 5 (Table 5). ROC curves were generated for the scoring system. The AUCs were 0.629 (95% CI 0.595–0.662) for all patients, 0.656 (95% CI: 0.614–0.699) for MetS patients and 0.569 (95% CI 0.517–0.621) for non-MetS patients. The difference in AUCs was not significant among the three groups (*P* = 0.223, Table 6, Fig. 3).

**Table 5:** Multivariable analysis of the MetS components

Variables	*OR*(95%CI)	*P* Value	Regression coefficeint	point
Elevated BMI	2.399 (1.656–3.476)	< 0.001	0.875	9
Elevated BP	1.840 (1.259–2.688)	0.002	0.610	6
Elevated FBG	1.301 (0.935–1.809)	0.118	0.263	3
Reduced HDL-C	1.098 (0.790–1.528)	0.578	0.094	1
Elevated TG	1.657 (1.230–2.233)	0.001	0.505	5

*BMI* body mass index, *BP* blood pressure, *FBG *Fasting blood glucose; *HDL-C* high-density lipoprotein cholesterol, *TG* Triglycerides,* OR* odd ratio

Adjusted for age, sex, heart rate, renal disease, former smoker, current smoker, prior myocardial infarction, stroke, history of percutaneous coronary intervention, white blood cell, homocysteine, platelet, creatinine, eGFR, glycated albumin, uric acid, ventricular ejection fraction; left ventricular end-diastolic dimension; left ventricular end-systolic dimension

**Table 6:** The ROC Curve analysis of the MetS with poor collateralization

Factors	AUC	P	95%CI	Se(%)	Sp(%)	Cut off point	P ^*^
All patients	0.629	< 0.001	*0.595–0.662*	*42.5%*	*77.2%*	13.5	0.223
MetS	0.656	< 0.001	*0.614–0.699*	*61.7%*	*61.7%*	14.5	
non-MetS	0.569	0.012	0.517–0.621	55.6%	58.1%	6.5	

Italic values indicate significance of *P * value (*P * < 0.05)

*Se *sensitive, *SP *specifity

P* The comparison among three groups

## Discussion

In this cohort study, we demonstrated significant MetS values in coronary collateral growth based on multivariable and subgroup analyses. After adjusting for confounding factors, MetS was also an independent factor of poor coronary collateralization. In addition, for MetS component analysis and model establishment, BMI was the strongest predictor of MetS components.

There are several diagnostic criteria of MetS, such as the National Cholesterol Education Program (NCEP) criteria, International Diabetes Federation (IDF) and The American Heart Association/National Heart, Lung, and Blood Institute (AHA/NHLBI). A cross-sectional survey of 109,551 Chinese adults has previously explored the best diagnostic criteria from the available definitions of MetS when assessing the risk of DM. After logistic regression analysis, the adjusted ORs are higher using the NCEP criteria (3.65, 3.52–3.79) than IDF (2.50, 2.41–2.60) and AHA/NHLBI (3.03, 2.92–3.24), indicating that the NCEP MetS definition may be more suitable for the assessment of DM risk in the Chinese population [17]. For cardiovascular disease, the prevalence of cardiovascular disease is more evident when MetS is defined according to the NCEP criteria (OR: 1.40) compared to AHA/NHLBI (OR: 1.34) and IDF (OR: 1.31) in the Chinese population [18]. Therefore, we selected the NCEP criteria, which may be more suitable in our study.

### Mechanisms of coronary collateralization reduction in MetS

The underlying molecular mechanisms of reduced CCs in MetS are poorly understood and are regulated by multiple factors. When a coronary artery is completely occluded, intra-arterial pressure gradients and shear stress on the arterial wall are increased, triggering inflammation, the redox state, gene expression and coronary collateral growth [19]. First, when an artery is occluded, vascular channels are stimulated by shear stress-driven redirection of flow. Then, neutrophils and lymphocytes activate a series of downstream molecular pathways, which increase the expression of proteins involved in monocyte attraction and adherence. Next, inflammatory cytokines adhere to the endothelium and differentiate into macrophages, which play an important role in vessel growth and development. Finally, collateral vessels increase in diameter because muscle cells proliferate and switch to a contractile phenotype [8].

The general characteristics of MetS are oxidative stress and endothelial dysfunction, which directly affect the vascular cell phenotype switch [20]. ROS are overproduced in MetS patients; ROS are the major cause of mitochondrial dysfunction, and they reduce the production of ATP. The reduction in ATP delays the phenotypic switch from quiescent to proliferating and migrating phenotypes [21]. With the persistent inflammatory state in MetS, the expression of proinflammatory cytokines is increased, triggering ROS production and reducing NO production, which play a vital role in oxidative stress and endothelial dysfunction [22]. In addition, these cytokines act as mediators to regulate signalling pathways of coronary collateralization growth, such as MAPK pathways and the Rho/Rho kinase pathway [23].

### Relationship between MetS components and coronary collateralization

MetS consists of five factors, namely, insulin resistance, abdominal obesity, HT, high TG levels and low HDL-C levels, which are indicated by the following four central features: insulin resistance, visceral obesity, atherogenic dyslipidaemia and endothelial dysfunction [24]. These factors are interrelated as well as independent. In obese patients, baroreceptor sensitivity is impaired, and activation of the sympathetic nervous system causes HT. In addition, obesity is a risk factor for T2DM and dyslipidaemia [25]. A previous study divided 104 obese patients into two groups, namely, poor and good collateralization, and demonstrated that BMI is higher in poor collateralization, which is negatively correlated with the Rentrop score [26]. In our study, BMI was an independent risk factor in the three study cohorts, and the regression coefficient was maximum. An increase in oxidative stress, aggravation of the inflammatory process and reduction in proangiogenic factors are the major causes of poor collateralization in T2DM patients [8]. The relationship between HT and CC remains controversial. Some researchers have found that high BP is positively related to a well-developed CC, which might increase perfusion and fluid shear stress [27]. Börekçi A et al. found that there is a negative relationship between CC and HT. We have previously found that SBP is an independent risk factor in all individuals [28]. Recent studies have demonstrated that T2DM, in particular, affects the early stages of collateral growth, whereas HT has an impact on later remodelling stages [29]. Dyslipidaemia plays an important role in the process of endothelial cell dysfunction and arterial stiffness, which are associated with coronary collateral vessel growth. HDL-C is positively associated with the angiographic collateral score in the Kadi study [30]. However, Shen Y et al. found no association between serum HDL-C level and coronary collateral score [31]. In the present study, there was no significant difference between HDL-C levels and CC. According to the regression coefficient of the components, the prognostic values were different. The sensitivity and specificity were increased by the scoring systems.

### Basic and clinical research

The present study demonstrated that MetS is robustly associated with poor CCs by logistic analysis. In rat models, CC growth is maximal at 9 days of coronary occlusion in healthy rats but does not occur in MetS rats. After administration of anti-miR-21 to block cell proliferation in MetS rats, CC growth was improved significantly. MetS influences the CC growth by blocking the vascular switch [32]. Hattan N et al. found that a reduction in oxidative stress improves CC growth. Compared to lean littermate rats, the ratio of collateral-dependent zone (CZ)/normal zone (NZ) flow is lower in MetS rats. After administering ecSOD and VEGF to reduce oxidative stress, the CZ/NZ ratio was increased [33]. A previous study has found that MMP12 acts as a novel positive regulator that inhibits coronary collateral development in a rat model of MetS. Compared to healthy rats, coronary collateral was reduced in MetS rats after left artery disease occlusion. After inhibition of MMP12 to block endostatin and angiostatin, the coronary collateral grew [34].

In clinical practice, Yilmaz MB et al. enrolled 596 consecutive patients with total occlusion of the right coronary artery. The presence of MetS was significantly higher in patients with poor CCs than in those with good CCs. After regression analysis, MetS was an independent predictor of angiographically determined poor CCs [35]. A previous observational study, which enrolled 387 patients with at least one coronary occlusion, has reported that AIP is independently associated with less developed collateral vessels. The multivariable analysis adjusted for potential confounding factors and indicated that MetS was also an independent risk factor [36]. The results were further confirmed in our study. Compared with clinical studies, the present study investigated the significance of MetS components by subgroup analysis. According to the ROC curve, the scoring models were targeted to different populations, including MetS patients and non-MetS patients, which will improve prediction efficacy and accuracy in clinical practice.

### Classical risk factors for coronary artery disease and coronary collateralization

Smoking is a traditional risk factor for coronary artery disease, which leads to endothelial dysfunction by increasing the production of reactive oxygen species and inducing atherosclerosis [37]. The risk factors for coronary collaterals are still controversial. Nicotine has been identified as a potent angiogenic agent through the endogenous nicotinic cholinergic pathway in endothelial cells, which is involved in physiological and pathological angiogenesis [38]. In addition, nicotine may also promote arteriogenesis, which may be mediated, in part, by activation of endothelial-monocyte interactions involved in arteriogenesis [39]. Bhatt H et al. enrolled 180 patients with CC and 383 individuals without CC and revealed a positive correlation between smoking and the presence of CC [40]. However, some researchers have found opposing evidence that smoking causes rarefaction of coronary collateral circulation [41]. In the present study, there were no significant differences in current smoking and former smoking. Due to the loss of detailed information, we did not analyse the relationship between the duration and density of smoking and the growth of CCs. Only one previous study in 2007 has reported on the duration and density of smoking in CC in which smoking was defined as past or current in 242 patients and pack years was categorized into never-smokers, < 10, 10–19, 20–29 and  ≥ 30 pack years. After analysis, current smoking was positively associated with the presence of coronary collateralization [42], but pack years of smoking was not associated with the presence of CC. This provides an intriguing direction for further research.

Obesity is an important component of MetS, and the waist/hip ratio or BMI represents obesity according to the WHO criteria. In NCEP ATP III, obesity is defined as waist  ≥ 102 cm (men) or ≥ 88 cm (women). Despite the alternatives for BMI, as an assessment method, BMI is the internationally accepted standard method used by researchers. Some research has found that BMI is positively associated with lower body subcutaneous adipose tissue mass and visceral adipose tissue accumulation. However, waist circumference is a powerful tool to measure abdominal fat [43]. A recent study has indicated that the combination of BMI and waist circumference identifies a high-risk obesity phenotype better than either measure alone [44]. Another previous study has reported no significant differences between BMI and waist circumference as obesity indices for cardiovascular risk factors, and BMI may be more strongly associated with hypertension than waist circumference [45].

## Limitations

Our study has some limitations. First, the single-centre nature of this study and the relatively small number of enrolled patients may have introduced selection bias. Second, the underlying mechanistic link between MetS and CC is not clear, and potential risks may affect CC growth and the MetS state. Even though subgroup analysis and multivariate logistic regression analysis were performed, it is uncertain whether we will be able to use the results for any recommendations. Furthermore, we used the Rentrop scoring system to identify the condition of CC due to the ease of assessment. However, coronary collaterals may be more accurately assessed by haemodynamic indices.

## Conclusions

In patients with chronic total occlusion, poor coronary collateralization is tightly associated with MetS, especially BMI and T2DM. After adjusting for potential risks and establishing a scoring system, we found that MetS is an independent risk factor for CC growth.

## Data Availability

Data available on request from the authors.
